# Recent Advances in Renewable Polymer Production from Lignin-Derived Aldehydes

**DOI:** 10.3390/polym13030364

**Published:** 2021-01-24

**Authors:** Nahyeon Lee, Yong Tae Kim, Jechan Lee

**Affiliations:** 1 Department of Energy Systems Research, Ajou University, 206 Worldcup-ro, Suwon 16499, Korea; skgus@ajou.ac.kr; 2 C1 Gas & Carbon Convergent Research Center, Korea Research Institute of Chemical Technology, 141 Gajeong-ro, Daejeon 34114, Korea; ytkim@krict.re.kr; 3 Department of Environmental and Safety Engineering, Ajou University, 206 Worldcup-ro, Suwon 16499, Korea

**Keywords:** aldehydes, biopolymer, biorefinery, lignin, lignocellulosic biomass, phenolics

## Abstract

Lignin directly derived from lignocellulosic biomass has been named a promising source of platform chemicals for the production of bio-based polymers. This review discusses potentially relevant routes to produce renewable aromatic aldehydes (e.g., syringaldehyde and vanillin) from lignin feedstocks (pre-isolated lignin or lignocellulose) that are used to synthesize a range of bio-based polymers. To do this, the processes to make aromatic aldehydes from lignin with their highest available yields are first presented. After that, the routes from such aldehydes to different polymers are explored. Challenges and perspectives of the production the lignin-derived renewable chemicals and polymers are also highlighted.

## 1. Introduction

Lignocellulosic biomass has been regarded as the renewable alternative carbon that potentially replaces fossil fuel resources [1,2,3,4,5]. It consists mainly of cellulose, hemicellulose, and lignin [6]. Lignin is a heterogeneous aromatic biopolymer, which comprises 15–30% of the weight of lignocellulosic biomass and an integral part of cell walls of terrestrial plants [7,8]. Lignin has aromatic structure; hence, it is considered a considerable renewable carbon source [9,10,11,12]. Nevertheless, the utilization of lignin has been limited to energy recovery. For example, most biorefinery processes degrade lignin, involving breakage of unstable ether bonds (e.g., β-O-4 linkage) and C–C bond formation of reactive intermediates via their condensation [13,14,15,16]. The resultant degraded lignin is usually incinerated for heat and power production because it is hard to be depolymerized [17,18]. In order to enhance sustainability and economics of biorefinery of using lignocellulosic biomass as a raw material, the effective utilization of lignin is crucial [19].

There have recently been efforts to search suitable renewable raw materials for polymer production. In particular, the development of bio-based polymers from lignin-derived monomers has gained great interest [20,21]. Within these contexts, this review provides up-to-date summary and knowledge of bio-based polymers synthesized from lignin-derived aromatic aldehyde compounds. To this end, we first discuss a variety of methods to convert lignin into aromatic aldehydes (i.e., syringaldehyde and vanillin). Various routes from these substances to bio-based polymers are then outlined. At last, present challenges and prospects of the processes are discussed.

## 2. Production of Aromatic Aldehydes from Lignin

**Figure 1:**
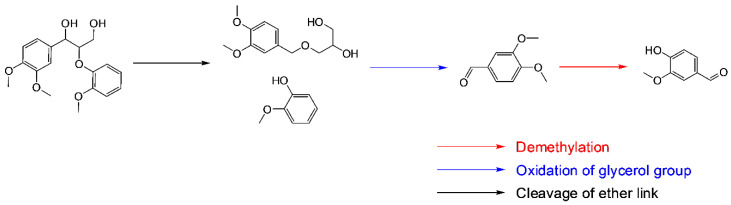
Potential reaction pathway for aerobic oxidation of lignin to vanillin via a series of reactions [37].

Aerobic oxidation of lignin under alkaline conditions selectively produces aromatic aldehydes such as syringaldehyde and vanillin [22]. Vanillin is commercially produced via alkaline aerobic oxidation of sulfonated lignin [23,24]. Alkaline oxidation of lignin has been extensively investigated mostly using condensed lignin feedstocks such as Kraft lignin [25,26,27,28,29] and sulfonated lignin [29,30,31,32,33]. For example, different research groups conducted studies into the effect of reaction parameters (e.g., temperature, O_2_ pressure, and NaOH concentration) on aerobic oxidation of Kraft lignin and sulfonated lignin [34,35,36]. Table 1 lists the results of alkaline aerobic oxidation of different lignins. Figure 1 illustrates reactions involved in aerobic oxidation of lignin to aromatic aldehydes (e.g., vanillin).

Earlier studies on alkaline aerobic lignin oxidation used lignins with highly modified structures (e.g., alkali-treated lignin, Kraft lignin, and lignosulfonates) [38,39,40] as the substrate. Such technical lignins have different structures and characteristics from native lignin because they change during delignification process, associated with delignification and biomass treatment and delignification methods. For example, Kraft lignin contains 1–3% sulfur, used to prepare resins, carbon fibers, and low-molecular-weight compounds. Organosolv lignin is sulfur-free and has many reactive sites, employed as additives to inks, paints, and coatings. Detailed comparison of characteristics of various technical lignin can be found in a recent review by Ekielski and Mishra [41].

In a recent study, a more native-like lignin (poplar sawdust containing 29.4 wt.% lignin) was used for the alkaline aerobic oxidation, as a surrogate substrate for an unmodified, highly reactive, and non-degraded lignin [42]. For this reaction, other than syringaldehyde and vanillin, syringic, *p*-hydroxybenzoic, and vanillic acids and acetosyringone were identified. During the oxidation of poplar lignin, the resultant syringaldehyde and vanillin were further oxidized to non-aromatic carboxylic acids. Vanillin was much less reactive for the formation of non-aromatic acids than syringaldehyde. High temperatures, high NaOH concentrations in the reactant, and short reaction times favored the production of syringaldehyde and vanillin. The reason why long reaction deteriorates the yield was the aromatic aldehydes degraded rapidly in the presence of oxygen. About 20 wt.% yield of syringaldehyde and vanillin was achieved with the poplar lignin at 175 °C and 0.5 MPa O_2_ under 2 M NaOH.

The work done by Schutyser et al. also investigated the effect of Cu-based catalysts (CuSO_4_ or LaMn_0.8_Cu_0.2_O_3_) on alkaline aerobic oxidation of the poplar lignin [42]. These catalysts did not enhance the maximum aromatic aldehyde yield; however, they made the high yield could be obtained at a wider range of reaction conditions. The findings were somewhat contradictory to those of other studies that used modified lignins. This may be because the native-like lignin is reactive enough without catalyst compared to modified ones. The roles and mechanisms of the catalyst in the oxidation of the lignin contained in native biomass could not be explained, remaining as future studies.

Impurities contained in lignin feedstocks (e.g., inorganics and carbohydrates) may hinder the isolation of vanillin and syringaldehyde. Therefore, such impurities need to be removed from lignin (even technical lignin) prior to its oxidation in order to make uniformly fractionated lignin. The use of uniformly fractionated lignin would be beneficial to enhance the yield of vanillin and syringaldehyde. Various approaches to effectively isolate and recover vanillin and syringaldehyde from the reaction stream are found in the studies done by several groups [24,43,44].

Although many studies on the aromatic monomer production from lignin have carried out, there has been no study into economic feasibility of the aldehyde production from lignin yet. A recent paper, however, has reported technoeconomic feasibility of a lignin-derived aromatic compound (e.g., catechol) [45]. The total cost investment of a plant which capacity is 2544 kg of feedstock per day was estimated to be approximately 4.9 million USD. The price of catechol production from lignin was calculated as 1100 USD per ton of catechol (a valorization ratio of 3.02). The estimation indicated that the lignin-derived catechol can be competitive in the market, while it was highly associated with assumptions on price of raw materials and sell price of products. A similar situation should be applied to a large-scale production of lignin-derived aromatic aldehydes.

**Table 1:** Alkaline aerobic oxidation of different lignin feedstocks.

Entry	Lignin Type	Product	Reaction	Catalyst	Reaction Conditions	Yield	Ref.
1	Swollen residual enzyme lignin	Vanillin, Syringaldehyde	Alkaline nitrobenzene oxidation	-	170 °C; 3 h	Vanillin: 13.39% Syringaldehyde: 14.19%	Wang et al. [46]
2	Double enzymatic lignin	Vanillin, Syringaldehyde	Alkaline nitrobenzene oxidation	-	170 °C; 3 h	Vanillin: 9.17% Syringaldehyde: 15.99%	Wang et al. [46]
3	Kraft lignin	Vanillin, Syringaldehyde	Alkaline nitrobenzene oxidation	-	170 °C; 3 h	Vanillin: 2.51% Syringaldehyde: 2.92%	Wang et al. [46]
4	Alkali lignin	Vanillin, Syringaldehyde	Alkaline nitrobenzene oxidation	-	170 °C; 3 h	Vanillin: 6.52% Syringaldehyde: 4.16%	Wang et al. [46]
5	Kraft lignin	Vanillin	Oxidation	-	120–125 °C; 10 bar; 130 min	3.5–7.6%	Araújo et al. [27]
6	Oil palm empty fruit bunches lignin	Vanillin	Nitrobenzene oxidation	-	165 °C; 3 h	1.6%	Mohamad Ibrahim et al. [47]
7	Lignosulfonates	Vanillin	Oxidation		185 °C; 11 atm; 85 min	7.2%	Bjørsvik and Minisci [31]
8	Kraft lignin	Vanillin	Chemical oxidation	-	133 °C; 2.8 bar O_2_; 35 min	10.8%	Fargues et al. [25]
9	Pine kraft lignin	Vanillin	Oxidation	-	120 °C; 5.5 bar N_2_, 9.3 bar O_2_; 60 min	10%	Mathias and Rodrigues [34]
10	Pine wood lignin	Vanillin	Oxidation	-	160 °C; 0.9 MPa O_2_; 20 min	23.1%	Taraban’ko et al. [48]
11	Poplar lignin	Vanillin, Syringaldehyde	Alkaline aerobic lignin oxidation		175 °C; 5 bar O_2_, 15 bar He; 10 min	Vanillin: 7% Syringaldehyde: 13%	Schutyser et al. [42]
12	Eucalyptus globulus dioxane lignin	Vanillin, Syringaldehyde	Nitrobenzene oxidation	-	170 °C; 4 h	Vanillin: 5.66% Syringaldehyde: 33.2%	Rodrigues Pinto et al. [28]
13	Lignosulfonates	Vanillin, Syringaldehyde	Oxidation	CuSO_4_	150 °C; 10 bar O_2_; 20 min; CuSO_4_ loading of 20%	Vanillin: 4.5% Syringaldehyde: 16.1%	Santos et al. [32]
14	Pine lignin	Vanillin, Syringaldehyde	Oxygen delignification	-	100 °C; 0.7 MPa O_2_; 60 min	Vanillin: 8.8% Syringaldehyde: 0.72%	Wong et al. [49]
15	Eucalyptus pulp lignin	Vanillin, Syringaldehyde	Oxygen delignification	-	100 °C; 0.7 MPa O_2_; 60 min	Vanillin: 4.62% Syringaldehyde: 7.85%	Wong et al. [49]
16	Cornstalk lignin	Syringaldehyde	Catalytic oxidation	LaFe_0.8_Cu_0.2_O_3_	120 °C; 5 bar O_2_; 30 min; LaFe_0.8_Cu_0.2_O_3_ loading of 5%	11.5%	Zhang et al. [50]
17	Softwood lignin	Vanillin	Alkaline wet oxidation	-	140 °C; 10 bar O_2_; 280 min	3%	Gomes and Rodrigues [51]
18	Dealkali lignin	Syringaldehyde	Catalytic oxidation	LaFe_0.2_Cu_0.8_O_3_	160 °C; 0.8 MPa O_2_; 2.5 h; LaFe_0.8_Cu_0.2_O_3_ loading of 5%	10%	Li et al. [52]
19	Kraft lignin	Vanillin	Alkaline wet oxidation	CuMn (1:3)	150 °C; atmospheric pressure; 60 min; CuMn loading of 0.2%	6.7%	Jeon et al. [53]
20	Native softwood lignin	Vanillin	Aerobic oxidation	-	120 °C; 72 h	21%	Maeda et al. [54]
21	Kraft lignin	Vanillin	Alkaline wet oxidation	-	140 °C; 10 bar O_2_; 2 h	4.3%	Gomes and Rodrigues [55]
22	Lignin from Kraft cooking liquor	Vanillin	Alkaline nitrobenzene oxidation	-	170 °C; 3 h,	3.9%	Gitaari et al. [56]
23	Kraft lignin	Vanillin	Oxidation	-	160 °C; 1 MPa O_2_; 1 h	21.1%	Zhu et al. [57]
24	Kraft lignin	Vanillin	Catalytic oxidation	CuSO_4_	140 °C; 1 h; CuSO_4_ loading of 10%	10.9%	Zhang et al. [58]
25	Organosolv lignin	Vanillin, Syringaldehyde	Electro-oxidation	-	Room temperature; 1 h	17.5%	Yan et al. [59]
26	Kraft lignin	Vanillin	Oxidative depolymerization	-	180–220 °C; 1–2 atm O_2_; 1–2 h	1.8–5.2%	Liu et al. [60]
27	Eucalyptus lignin	Vanillin, Syringaldehyde	Alkaline nitrobenzene oxidation	-	170 °C; 4 h	Vanillin: 7.3% Syringaldehyde: 29.3%	Ninomiya et al. [61]

## 3. Production of Polymers from the Lignin-Derived Aromatic Aldehydes

### 3.1. Production of Vanillin- and Syringaldehyde-Derived Polymers

**Figure 2:**
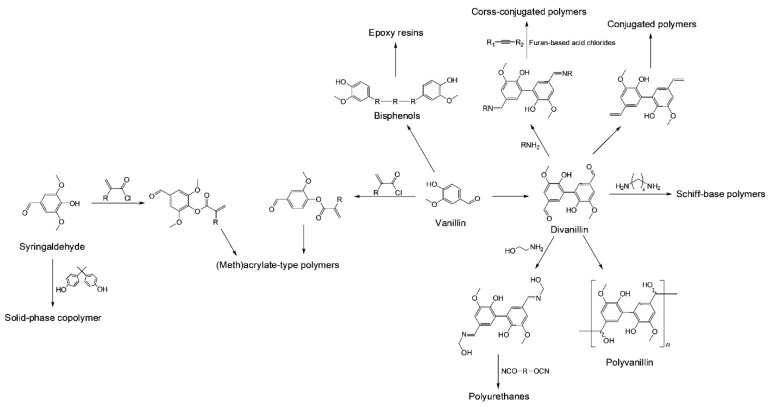
Various polymers that can originate from vanillin or syringaldehyde.

Lignin can be directly employed to produce various polymers such as polyesters, polyurethanes, and resins [62,63]. The polymers are synthesized directly from lignin via functionalization of hydroxyl groups in lignin structure, or lignin is utilized as blends, copolymers, and composites [64,65]. However, the direct use of lignin for the polymer production has several limitations. For instance, properties of the resultant polymers are highly dependent upon the composition and structure of lignin [66]; therefore, it is challenging to control the properties. In addition, lignin often comprises composites or blends as in minor amounts, and their major portions still originate from petroleum. Compared to lignin itself, lignin-derived monomers such as syringaldehyde and vanillin (discussed in Section 2) have relatively well-defined aromatic structure [65]. This aromatic structure gives important polymer properties like hydrophobicity, rigidity, and resistance to fire. In this section, it will be given an overview of methods employed to make different polymers from the lignin-derived syringaldehyde and vanillin. Figure 2 depicts various polymers that can originate from vanillin or syringaldehyde.

Among a wide range of aromatic monomers can be derived from lignin [67], only vanillin production is currently industrialized, which makes vanillin a particularly attractive monomer for polymer synthesis [63,68]. In earlier applications, vanillin is commonly functionalized to make epoxy resins [69,70,71] by adding a second alcohol [72] and used to make polymeric composite materials [73,74] and resins [75,76,77]. More recently, divanillin is used as a polymer precursor. Divanillin is readily made via enzymatic oxidative dimerization of vanillin on horseradish peroxidase (*Armoracia rusticana*) [78]. Alternatively, it can be produced oxidative coupling by using laccase [79,80,81], iron(III) chloride [82], and persulfate salts [83,84,85]. For instance, Fang et al. has very recently showed that sodium persulfate (Na_2_S_2_O_8_) is an effective persulfate salt for the synthesis of divanillin due to its high water solubility and inexpensiveness [85]. It could also be electrochemically synthesized [86]. No structural space and short segments in divanillin lead to minimizing rotational motion of its backbone, resulting in dynamic performance of polymeric networks [81,87].

Divanillin has been applied to the synthesis of various polymers including polyvanillin [83,86], Schiff base polymers [88], epoxy thermosets [81,89,90], and lignin oligomers [91]. For example, reaction between divanillin and alkyl diamines in the presence of ethanol followed by reflux resulted in Schiff base polymers having the degree of polymerization ranging from 25 to 32 [88]. Divanillin could also be transformed to α,ω-dienes that were further converted into a conjugated polymer through acyclic diene metathesis [92]. Divanillin was employed as a chain extender to synthesize polyurethanes with a modification with ethanolamine [93]. Divanillin was able to electrochemically polymerized to polyvanillin considered a functionalized renewable polymer [86].

Bisphenols are precursors of a wide variety of polymers including polyesters [79], polycyanurates [72], polycarbonates [72], and epoxy resins [85,94] that are made through simple modifications of the structure of bisphenols. Two strategies have been available to make bisphenols from vanillin. First one involves coupling reactions between aromatic rings of two phenolic compounds through electrophilic condensation [95] or enzymatic dimerization [79]. Second one is to functionalize side chain of the phenolic species, involving coupling of two phenolics via a cross-linker [94] and the generation of stilbenes [72]. Song and co-workers enzymatically co-polymerized syringaldehyde with petro-derived bisphenol A using a peroxidase (*Coprinus cinereus*) [96].

Polymers synthesized by modifying side chain of vanillin or syringaldehyde have also been reported. Rostagno et al. prepared different polyvinyl aromatic acetals by the condensation of poly(vinyl alcohol) and lignin-derived aromatic aldehydes such as syringaldehyde and vanillin [97]. Polymeric vanillin prodrug-based nanoparticles were synthesized by Kwon et al. [98], which are potentially applied to drug delivery systems and antioxidant therapeutics. Liu et al. derived acrylamides from vanillin via a three-step process which could be polymerized through free radical polymerization [99]. The free radical polymerization could also be used to prepare acrylate polymers from syringaldehyde and vanillin [100].

There have been efforts to expand the application of renewable polymers derived from vanillin and syringaldehyde. For instance, Kakuchi and co-workers have recently exploited the Kabachnik–Fields reaction as a method to post-modify vanillin or syringaldehyde-derived polymer, considering that aldehydes are important reactants in multi-component reactions [101]. The Kabachnik–Fields multi-component reaction between phosphites, amines, and polymers derived from vanillin or syringaldehyde completely changed aldehyde functionalities of the polymers to α-amino phosphonate functionalities.

An interesting use of renewable lignin-derived monomer was reported by Zhang et al. They synthesized polymeric porous microspheres from vanillin [102]. Vanillin first reacted with methacryloyl chloride, which made vanillin methacrylate. Suspension polymerization of the vanillin-derived monomer under aqueous phase resulted in microspheres with a >90 wt.% yield. The microspheres had surface porosity by N_2_-bubbling and optimizing the co-solvent ratio. When the aldehyde functionalities of the microspheres reacted with glycine, the microspheres were chelated by Schiff base. The chelating microspheres showed a high performance of Cu^2+^ (as a heavy metal surrogate) adsorption (135 mg g^−1^). Later on, the same researcher synthesized similar magnetic microspheres composed of polymethacrylate/Fe_3_O_4_ nanoparticles [103]. The magnetic moiety allowed an easy separation of the material from liquid media after its use. The microspheres were effective at adsorbing *p*-anisidine, proving its suitability for enzyme immobilization.

### 3.2. Properties of Vanillin- and Syringaldehyde-Derived Polymers

Thermoset polymers are used in many applications including coatings, packaging, adhesives, composites, and electronics because of their high strength, high, modulus, and good resistance to heat and chemicals [104]. The properties of thermosets can readily be varied, dependent upon molecular weight, nature of the polymers, and adjustable cross-linking density [105]. Of various classes of thermosets, epoxy resins represent approximately 70% of thermoset market owing to their superior mechanical and adhesive properties and high thermal chemical resistance [106].

The lignin-derived vanillin and syringaldehyde (as discussed in Section 2) can be used to make epoxy thermosets. For example, Zhao and Abu-Omar made triphenylmethane-type polyphenols from syringaldehyde and vanillin [107]. The polyphenols were then reacted with or without linoleic acid (as a plasticizer), followed by epoxidation and curing to obtain a final form of the resin. The use of linoleic acid decreased glass transition temperature from 167 to 82 °C and storage modulus from 12.3 to 3.6 GPa (entry 1 in Table 2).

Zhu and co-workers synthesized vanillin-based epoxy resins having flame retardant properties attributed to phosphorus functionalized by using *p*-phenylenediamine or 4,4-diaminodiphenylmethane as coupling agent [94]. The procedure led to epoxy resins with thermal and mechanical properties that are comparable to commercial diglycidyl ether of bisphenol A (DGEBA) (entry 2 in Table 2).

Thermoplastic polymers are applied to manufacturing packaging, fibers, furniture, insulators, containers, medical equipment, automotive parts, et cetera. In addition to epoxy thermosets, thermoplastic polymers can be derived from vanillin and syringaldehyde. For instance, different methacrylate polymers with high glass-transition temperatures were derived from vanillin and syringaldehyde. Acrylate- and methacrylate-type monomers were first made, including vanillin acrylate, vanillin methacrylate, syringaldehyde acrylate, and syringaldehyde methacrylate [100]. Free radical polymerization of such monomers yielded their corresponding polymers. The polymers (95–180 °C) had a lot higher glass transition temperature than poly (methyl methacrylate) (PMMA), polystyrene (PS), and polylactic acid (PLA) (48–110 °C) (entry 4 in Table 2). They also had higher initial degradation temperature ranging from 300 to 320 °C than PMMA (280 °C) and PLA (296 °C). Kim group used a dimerized vanillin to partially replace traditionally used chain extender (e.g., 1,4-butanediol) to make polyurethanes [93]. The resultant polymeric materials that containing divanillin-ethanol amine conjugate had enhanced Young’s modulus (8.0–9.7 MPa) and strain (644.8–770.9%) than those of typical polyurethane (7.5 MPa and 522.6%, respectively) (entry 9 in Table 2).

Kayser et al. reported a cross-conjugated pyrrole-based fluorescent polymer comprising biomass-derived monomers such as vanillin and furan-based acid chlorides for the first time [108]. Multi-component polymerization substituted by catechyls and mediated by phosphonites was used to make the cross-conjugated polymer using light alkenes (or alkynes) and diacid chlorides. Investigation into the polymer’s optical properties (e.g., fluorescence and UV-vis absorbance) proved that the polymer is blue-emitting, and the emission can be modulated by modifying its structure. The authors of this study stated that the polymer is potentially applied to produce polymer-based light emitting diode. Properties of more vanillin- and syringaldehyde-derived polymers are also available in Table 2.

**Table 2:** Thermomechanical properties of vanillin- and syringaldehyde-derived polymers.

Entry	Monomers	Polymerization Method	Final Polymer	Glass Transition Temperature (°C)	Degradation Temperature (°C)	Other Properties	Ref.
1	Vanillin, syringaldehyde	Epoxidation; curing at 80 °C for 8 h	Epoxy resin	82–167 ^a^	*T*_5%_ = 220–269 ^b^	-	Zhao et al. [107]
2	Vanillin	Epoxidation; curing at 160 °C for 2 h	Epoxy resin	166–214 ^c^	*T*_5%_ = 286–356 ^d^	-	Wang et al. [94]
3	Vanillin methacrylate	Suspension polymerization; curing at 65 °C for 6 h	Polyvanillin methacrylate	102 ^c^	Degradation *T* range = 250–480 ^b^	-	Zhang et al. [102]
4	Vanillin, syringaldehyde	Free radical polymerization	(Meth)acrylate-type polymers	95–180 ^c^	*T*_max_ = 340–360 ^b^	*M*_n_ = 7600–14,600 g mol^−1^; Đ = 1.89–4.07	Zhou et al. [100]
5	Vanillin	Electrochemical reductive polymerization	Polyvanillin	-	*T*_50%_ = 440 ^d^	*M*_n_ = 9850–11,784 g mol^−1^; Đ = 1.42–1.58	Amarasekara et al. [86]
6	Vanillin	Reversible addition-fragmentation chain-transfer (RAFT) polymerization	Methacrylate-type polymers	111–139	*T*_max_ = 281–327	*M*_n_ = 15,000–41,000 g mol^−1^; Đ = 1.12–1.39	Holmberg et al. [109]
7	Syringyl methacrylate	RAFT polymerization	Poly(syringyl methacrylate)	114–205 ^e^	Initial degradation *T* = 256–303 ^d^	*M*_n_ = 11000–38,000 g mol^−1^; Đ = 1.32–1.74	Holmberg et al. [110]
8	Vanillin-derived bis-benzoxazine monomer	Ring opening polymerization	Poly(bisbenzoxazine)	202–255 ^e^	Degradation *T* range = 220–450 ^b^	-	Sini et al. [111]
9	Vanillin	-	Divanillin-ethanol amine conjugate-based polyurethane	−68.1 to −67.2 ^c^	*T*_5%_ = 329.6–341.5 ^b^	-	Gang et al. [93]
10	Vanillin, syringaldehyde, etc.	-	Polyvinyl acetals	114–157	*T*_95%_ = 185–308 ^b^	*M*_n_ = 22,300–46,000 g mol^−1^	Rostagno et al. [97]
11	Vanillin	-	Poly (ether benzoxazole)	-	>400 ^b^	-	Sun et al. [112]
12	Vanillin-based monomers	Phosphonite-mediated multicomponent polymerization	Fluorescent polymers	-	-	*M*_n_ = 3000–12,700 g mol^−1^; Đ = 1.8–2.3	Kayser et al. [108]
13	Vanillyl alcohol	-	Polyurethane	59	*T*_5%_ = 178 ^b^	*M_w_* = 32,000 g mol^−1^	Tachibana and Abe [113]
14	Vanillin	-	Polyurethane (PU-3)	80.4 ^c^	*T*_5%_ = 229 ^b^	*M*_n_ = 4000 g mol^−1^	Zhao et al. [114]
15	Vanillin	-	Cured epoxy resins	-	*T*_5%_ = 394 ^b^	*-*	Shibata and Ohkita [115]
16	Vanillin	Phthalonitrile functionalization	Phthalonitrile resins	>500 ^c^	*T*_5%_ = 477–482 ^b^	*-*	Han et al. [116]
17	Hydrovanilloin	Electrochemical dimerization	Hydrovanilloin—Diglycidyl Ether Phenoxy Resin	135	*T* = 255 ^d^	*-*	Amarasekara et al. [117]
18	Vanillin methacrylate	Free radical solution homo-polymerization	Vanillin-derived polymer (PVMA)	-	-	*M*_n_ = 17,900 g mol^−1^	Zhao et al. [118]

a By dynamic mechanical analysis; b By thermogravimetric analysis (TGA) in N2; c By differential scanning calorimetry (DSC) in N2; d By TGA in air; e By DSC in air.

## 4. Summary and Outlook

Lignin is an intriguing aromatic biopolymer. The release of more chemicals derived from lignin can expand available biorefinery feedstocks, thereby making economics of biorefineries and pulp and paper industries profitable. In this regard, this review gives an overview of processes that enable to deliver bio-based polymers from pre-isolated lignin or lignocellulose in good selectivities. As shown in this review, the applications of lignin to the manufacture of bio-based polymers demonstrate why lignin is regarded as a key renewable source in biorefineries. However, there are still challenges that need to be solved to be capable of the full implementation of lignin as renewable feedstock for bio-based polymers.

There are a large number of strategies to transform lignin into aromatic aldehydes such as syringaldehyde and vanillin. Many studies have recognized that re-condensation reactions occurring during processing lignocellulosic biomass and lignin conversion processes have pronounced effects on the product yields. Efforts to suppress the undesired reactions have been made. For instance, the introduction of formaldehyde to isolating lignin from lignocellulosic biomass achieved the yields at a near theoretical maximum [119]. Stabilization of reactive intermediate species during acidolysis of organosolv lignin was also reported [120,121,122]. Reductive catalytic fractionation led to high selectivities toward defined target products rather than obtaining a complex mixture of products [123].

The aerobic oxidation of lignin reported so far needs for high amounts of oxygen and alkali medium. However, most studies have still focused on increasing yields of target products, although reducing the oxygen and alkali consumption is clearly attention-worthy issue [124]. More efforts should be made to figure out how to lower the consumption of oxygen and alkali during the aromatic aldehyde production.

Most studies into the production of aromatic aldehydes from lignin that are currently available have laid emphasis on solving fundamental challenges of selective β-O-4 bond cleavage in lignin. However, cleavage of other linkages in lignin needs to be more actively investigated to fully valorize all lignin components. Stable and reusable catalysts that are tolerant to impurities contained in lignin should be developed [125]. Upscaling of experiments on converting lignin into the monomers is also required as an effort to make the lignin valorization process more economically viable.

For the conversion of lignin into aromatic aldehyde monomers, it is difficult to make a direct comparison of the conversion processes because characteristics and properties of lignin substances (e.g., purity, solubility, molecular weight, and β-O-4 content) are different, dependent upon the methods of lignin isolation and processing developed by different research groups. Thus, standardization of reporting performance of the lignin conversion methods is required. Regarding the standardization, the lack of standard lignin samples that have reproducible quality is a serious challenge. It is also necessary to standardize existing techniques for analysis both of the structure of lignin and lignocellulose and of lignin-derived complicated product mixtures.

Considerable advancements have come with polymers made from lignin-derived aromatic aldehydes. Many research groups have used pure and well-defined lignin substrates available from commercial sources to synthesize bio-based polymers, particularly thermoplastics and thermosets with comparable properties to conventional materials. However, there is still a discrepancy between chemical compounds frequently obtained from depolymerization of lignin and monomers most frequently used for the production of bio-based polymers. Novel methods to make the actual monomers with high yields are needed, which can open up possibilities of synthesizing emerging bio-based polymers having desired properties.

Catalytic conversions of lignin typically result in mixtures of different aromatic compounds that are directly employed to synthesize bio-based polymers. This can allow to avoid additional purification or separation steps, if reproducibility associated with the kind of lignin feedstocks and the composition of the aromatic mixtures is clarified. Moreover, the lignin-derived bio-based polymers are not degradable in nature; hence, their biodegradability and toxicity in the environment need to be further investigated.

In the past, the use of lignin as a starting material for the production of other chemicals was skeptical because the breakage of recalcitrant structure of lignin was a daunting task. Today, considerable advancements in research into biorefinery and catalysis make it possible the production of lignin-derived platform chemicals in acceptable yields. However, the full implementation of lignin as renewable feedstock for bio-based polymers still requires active collaborations across multiple disciplines and a constructive dialogue between people working in academia and industry. Constant efforts to develop lignin valorization processes will be essential for further innovation in biorefinery technologies.

## Data Availability

Not Applicable.
